# A Systematic Review of the Role of Oxytocin, Cortisol, and Testosterone in Facial Emotional Processing

**DOI:** 10.3390/biology10121334

**Published:** 2021-12-15

**Authors:** Ángel Romero-Martínez, Carolina Sarrate-Costa, Luis Moya-Albiol

**Affiliations:** Department of Psychobiology, University of Valencia, Avenida Blasco Ibañez, 21, 46010 Valencia, Spain; Carolina.Sarrate@uv.es (C.S.-C.); Luis.Moya@uv.es (L.M.-A.)

**Keywords:** cortisol, facial emotion processing, hormone administration, oxytocin, testosterone

## Abstract

**Simple Summary:**

The scientific community has paid special attention to facial emotional expression due to its importance in human surveillance as a communication tool. Humans need decoding abilities to understand the meaning of facial expressions and act accordingly. This ability is partly regulated by biochemical signals such as hormones, and it is of growing interest in understanding the role played by specific hormones such as oxytocin, cortisol, and testosterone. To date, there is a gap in the scientific literature summarizing how the manipulation of endogenous levels of oxytocin, cortisol, and testosterone through the exogenous administration of these hormones affects the processing of facial emotional expressions during adulthood in healthy and clinical populations of both genders. Therefore, we conducted a systematic review to summarize the evidence about how these three hormones influence facial emotional processing, paying special attention to studies that employed robust research designs (e.g., randomized, single- or double-blind, and/or placebo-controlled). The results obtained did not present a consistent pattern of association between the variables. In any case, these hormones slightly influenced facial emotion processing, but it is obviously extremely difficult to establish a direct association. To correctly understand the hormones’ influence, it is necessary to consider other factors such as the emotional valence and the participants’ gender, among others, which played an important role.

**Abstract:**

A topic of interest is the way decoding and interpreting facial emotional expressions can lead to mutual understanding. Facial emotional expression is a basic source of information that guarantees the functioning of other higher cognitive processes (e.g., empathy, cooperativity, prosociality, or decision-making, among others). In this regard, hormones such as oxytocin, cortisol, and/or testosterone have been found to be important in modifying facial emotion processing. In fact, brain structures that participate in facial emotion processing have been shown to be rich in receptors for these hormones. Nonetheless, much of this research has been based on correlational designs. In recent years, a growing number of researchers have tried to carry out controlled laboratory manipulation of these hormones by administering synthetic forms of these hormones. The main objective of this study was to carry out a systematic review of studies that assess whether manipulation of these three hormones effectively promotes significant alterations in facial emotional processing. To carry out this review, PRISMA quality criteria for reviews were followed, using the following digital databases: PsycINFO, PubMed, Dialnet, Psicodoc, Web of Knowledge, and the Cochrane Library, and focusing on manuscripts with a robust research design (e.g., randomized, single- or double-blind, and/or placebo-controlled) to increase the value of this systematic review. An initial identification of 6340 abstracts and retrieval of 910 full texts led to the final inclusion of 101 papers that met all the inclusion criteria. Only about 18% of the manuscripts included reported a direct effect of hormone manipulation. In fact, emotional accuracy seemed to be enhanced after oxytocin increases, but it diminished when cortisol and/or testosterone increased. Nonetheless, when emotional valence and participants’ gender were included, hormonal manipulation reached significance (in around 53% of the articles). In fact, these studies offered a heterogeneous pattern in the way these hormones altered speed processing, attention, and memory. This study reinforces the idea that these hormones are important, but not the main modulators of facial emotion processing. As our comprehension of hormonal effects on emotional processing improves, the potential to design good treatments to improve this ability will be greater.

## 1. Introduction

There is a classical philosophical interest in understanding the socialization role of human facial emotional expressions, but this phenomenon was not systematically studied until Charles Darwin published his book “*The Expression of Emotions in Man and Animals*”. He proposed that facial expressions of emotions are the key to humans’ surveillance as a communication tool because they help species to deal with life challenges [1]. He assumed that their genetic transmission is involuntarily produced, and that facial emotion expressions seem to be common across all cultures. Nonetheless, the genetic basis of facial emotion expressions was severely criticized, and the existence of cultural differences in facial expressions has been demonstrated [2,3]. Therefore, the heritability of this ability has been relativized, with the role of learning and experience being included as modulators of facial expressions of emotions. Accordingly, current explanations include the importance of heritability, as well as the modification of these expressions based on social learning processes [4].

In any case, it is undeniable that this form of nonverbal communication is extremely important for social cognition because it offers information to the rest of the community members about individuals’ inner states, and it tends to emerge a few days after birth [5]. Social interactions entail exchanging information to achieve mutual understanding among several participants. This dynamic system involves the transmission (emissary) and decoding of information (receptor), which impacts the subsequent behaviour of the individuals involved in this communicative dynamic. For nonverbal processing (e.g., emotional facial expression), the neurological system has to accurately decode facial expressions. In this regard, there is a growing body of scientific literature showing that higher social competence tends to be directly related to better decoding abilities [6,7]. Furthermore, alterations and/or deficits in the processing and recognition of emotional states in others are related to many psychiatric disorders, such as schizophrenia, anxious-depressed, and unsocialized-aggressive groups, among others [8,9,10].

There is a consensus among researchers about the importance of the amygdala in processing emotional stimuli, including emotional facial expressions. Not surprisingly (due their importance in surveillance), faces are stimuli that consistently elicit amygdala activation [11]. Researchers suggest that the amygdala tends to be consistently and strongly activated in positive and negative facial expressions of emotion [11,12,13]. Hence, it is important to understand how the amygdala interacts with other brain structures (cortical and subcortical) to process and discriminate facial expressions. For example, the amygdala seems to maintain a reciprocal and inverse relationship with the prefrontal cortex (PFC) to keep the balance in emotional processing [14,15]. In fact, the ventromedial PFC (vmPFC) particularly increases its activation in the presence of happy faces, but the dorsomedial prefrontal cortex (dmPFC) presents higher activation in response to negative facial expressions in comparison with neutral and happy expressions [16]. Obviously, these are only a few structures in a complex neurological system that includes multiple brain structures [17].

To understand facial emotion processing, not only is it important to pay attention to the activation of specific brain structures, but it is also necessary to study biochemical brain pathways. In this regard, some of the above-mentioned brain structures present differential sensitivity to specific chemical signals (e.g., hormones) [18,19,20]. Three of these hormones, known as oxytocin, cortisol, and testosterone, aroused great interest in the scientific community because it seemed that endogenous fluctuations in these hormones influence emotional processing in humans, [21,22]. Accordingly, increases or decreases in these hormones stimulate hormonal brain receptors, which, in turn, modulate, for example, the salience of certain emotional cues and affect the speed in detecting emotional stimuli. Nonetheless, a large number of these studies in humans have been correlational, whereas less is known about the existence of a causal relationship between manipulation of these hormones during adulthood and their role in facial emotional processing.

To study the relationship between these hormones and emotional processing, it is important to clearly establish the pathways for administering these hormones and, subsequently, modify endogenous levels interfering in cognitive processes. These pathways are particularly important because they depend on the percentage of hormone administered that would reach the central nervous system and influence facial emotional processing. For example, it has been highlighted that the intranasal administration of oxytocin is more appropriate than intravenous administration because it is easier and less invasive than the intravenous method. Through stimulation of the olfactory neurons in the olfactory epithelium with a spray, oxytocin would be transported to the olfactory bulb through olfactory or trigeminal nerves. From this point, it would be distributed through passive diffusion into the cerebrospinal fluid to reach brain structures, including those that manage facial emotional processing [23,24,25].

With all this in mind, the main objective of this study is to summarize, through a systematic review of the literature, how the manipulation of endogenous levels of oxytocin, cortisol, and testosterone through the exogenous administration of these hormones affects the processing of facial emotional expressions during adulthood in healthy and clinical populations of both genders, which has not been performed to date. Particularly, we analyse several processes that are closely related to facial emotional processing, such as accuracy in facial recognition, reaction times, rating arousal, attention and/or memory performance, rating trustworthiness/friendliness, and dominance/hostility. Finally, considering the existing data so far, we build a model to analyse the possible interactions between these variables and how they affect facial emotional processing, which, in turn, affects subsequent behaviours, such as prosocial and antisocial behaviours, in healthy and clinical populations of both genders. Moreover, the conclusions derived from this systematic review would also guide future research in this field and help to develop more robust research designs.

## 2. Method

### Search Strategy

We conducted this systematic review following the PRISMA (Preferred Reporting Items for Systematic Reviews and Meta-Analyses) guidelines. Hence, a literature search was performed in the following databases: PsycINFO, PubMed, Dialnet, Psicodoc, Web of Knowledge, and the Cochrane Library. Moreover, we also completed this process with hand-searching. All these processes were carried out from January to April 2020. Regarding manuscript selection, we specifically selected manuscripts with a robust research design (e.g., randomized, single- or double-blind, and/or placebo-controlled) to increase the value of this systematic review.

We started an initial search with broad terms, such as “hormonal”, “processing”, and “facial processing”, but this or a similar search produced redundant information. Afterwards, we specified several hormones, such as vasopressin, progesterone, prolactin, and oestrogens, along with the hormones finally included, but there were not enough controlled designs that assessed facial emotional processing. Therefore, we finally decided to establish that the best search strings, from our point of view, for this field of research and applied to the databases were: [(testosterone), OR (cortisol) OR (oxytocin), AND [(faces) OR (faces emotion) OR (faces expression)]].

All the papers finally selected for inclusion in this review met the following criteria: (a) they were empirical studies with humans; (b) the manuscripts had been peer-reviewed (avoiding congress abstracts or thesis); (c) they exclusively employed human adult faces (e.g., excluding computerized avatars or cartoons) or specific parts of adult faces (e.g., eyes); (d) participants were adults from healthy and/or clinical populations (e.g., schizophrenia, anxiety disorders, among others); (e) they assessed the association between hormonal values and emotional facial processing (e.g., accuracy, reaction times, attention, memory, etc.); (f) they were placebo-controlled; and, (g) they were written in English or Spanish.

We think it is particularly important to highlight that several manuscripts considered significance to be slightly higher than 0.05. However, to be rigorous, and considering the limited sample size in these studies, we employed a conservative criterion and decided that these results were not significant.

Two of the three authors carried out independent systematic reviews. Luckily, both authors agreed on 99% of the manuscripts considered for the present study. Only a few cases were discussed, and after providing evidence of their adherence to the inclusion criteria, both authors decided to include these manuscripts.

## 3. Results

**Figure 1:**
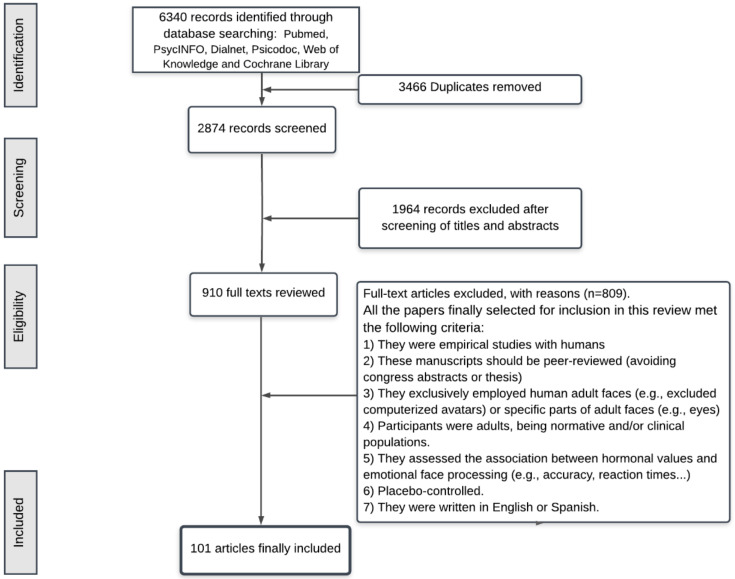
Flowchart.

As the flowchart shows (Figure 1), we finally included 101 articles in our systematic review. Unfortunately, a different number of manuscripts assessed each hormone. For example, most of them assessed oxytocin effects (72), followed by cortisol (16) and testosterone (13).

Regarding the presentation of the results, we initially provided a main summary of the studies’ characteristics (detailed in a table for each hormone). Afterwards, we provided the main results for each study. In this regard, each table states whether or not the study obtained a significant main effect. Given that the majority did not report an initial significance, we also added which interactions between independent variables guaranteed significance, as well as their effect size.

### 3.1. Oxytocin

We included a total of 72 studies in this review, and the sample sizes of these studies ranged from 16 to 120 participants. Even though we found that participants’ ages ranged from 18 to 90 years old, most of the studies recruited younger adults (20–45 years) and, furthermore, were mostly based on healthy individuals (66%). Regarding gender distribution, the majority of the studies only included men, followed by studies combining men and women. Finally, 10% of the studies based their conclusions mainly on groups of women. All of the studies included intranasal administration of oxytocin (Table 1).

Of all the studies included, only 18% reported a significant main effect of hormone administration on emotional facial processing [27,30,38,56,60,61,69,70,71,73,75,87,93]. Furthermore, the percentage of manuscripts that reached significance after including additional variables (e.g., emotional valence, gender, age, among others) combined with oxytocin (drug) administration was approximately 54% (Table 2).

Focusing on the healthy population, after hormone administration, results indicated that participants in the hormone/drug group showed higher accuracy in recognizing emotional faces, regardless of the emotional valence [30,38,56,60,61,70,73,75,87], but Cardoso et al. [27] found that oxytocin reduces accuracy. Moreover, this effect was more pronounced in men than in women, particularly in older men [26]. However, whether they focused on the emotional valence of faces or other variables related to the participants or the task (e.g., participants’ gender, age, dosage, among others), the conclusions were far from homogeneous.

Whereas a few studies concluded that oxytocin only enhanced recognition of neutral, surprised, and/or happy facial expressions [29,48,53,61,62,70,72], other researchers pointed out that oxytocin reduced accuracy in discriminating fearful faces in both genders [48,49,84]. Nevertheless, other studies concluded that, in men, oxytocin administration decreased accuracy for angry faces in men [49], and another study concluded that drug administration increased accuracy for angry faces only in women [72]. Furthermore, a previous study concluded that oxytocin enhanced accuracy in discriminating fearful faces only in men [39]. Lastly, it is particularly interesting that when the authors mixed facial emotional stimuli with incongruent emotional contexts (e.g., body position, scenes…), participants who received oxytocin only discriminated disgusted faces better in an anger context [54].

Focusing on clinical populations, a study reported that the administration of 10 IU of oxytocin in schizophrenic patients of both genders diminished facial emotional processing accuracy, but a dosage of 20 IU improved accuracy in recognizing facial emotions [85]. In this regard, research also demonstrated that individuals with antisocial personality disorder presented a worse baseline ability to recognize happy and fearful faces than the healthy population, but after oxytocin administration, their accuracy improved, and their performance did not differ from the control group [96]. Conversely, women who reported low levels of love withdrawal during childhood and who received a drug dosage presented reduced accuracy in general facial emotional processing [59].

It is important to assess not only the emotional valence of the stimuli, but also their level of difficulty. In this regard, a manuscript pointed out that participants’ ability to discriminate faces only improved after oxytocin administration on items with a high level of difficulty [30,97]. Conversely, Guastella et al. [87] and Mitchell et al. [91] found that oxytocin administration improved the ability to decode easy items, but this result was only reported in participants under the age of 16 [87]. Therefore, it is important to consider not only the level of difficulty, but also the age of the participants.

Oxytocin also affected the way participants rated the intensity or arousal of emotional faces. A study that combined healthy participants of both genders concluded that, after oxytocin administration, the intensity of facial emotions increased in both groups, but accuracy in recognizing the emotional valence diminished [27]. A posterior study extended these results. Specifically, Spengler et al. [65] pointed out that men who received oxytocin rated faces that expressed low-intensity happiness or fear as neutral. That is, oxytocin reduced their accuracy, but only on facial emotions with low intensity. Conversely, Quintana et al. [57] concluded that men who received oxytocin rated ambiguous faces with lower intensity only in the case of anger, compared to a placebo condition [57]. The rest of the studies failed to report significant results for changes in rating the intensity or arousal of facial emotional expressions [31,50,63].

The aforementioned results were for healthy participants. In clinical populations, oxytocin administration increased the perceived intensity of all six emotions in polydipsic patients [85]. Additionally, men with SAD perceived happiness of ambiguous faces with higher intensity after receiving oxytocin, in comparison with a placebo condition [94].

Regarding the rating of the trustworthiness and friendliness of emotional faces, whereas two studies concluded that oxytocin administration diminished the level of trustworthiness in angry faces for women and men [37,68], another study revealed that men rated angry faces with higher levels of trustworthiness than in the placebo condition, but this effect was not observed in women [51]. Moreover, another study concluded that, after drug administration, men rated neutral faces with lower levels of trustworthiness than in a placebo condition, whereas women receiving oxytocin rated neutral faces with higher levels of trustworthiness than those receiving a placebo [45]. Nevertheless, Quintana et al. [57] did not find significant changes in the level of trustworthiness in angry and happy faces after oxytocin administration in men.

The evaluation of the perceived rate of dominance in facial emotional expressions in Teed et al. [66] allowed them to conclude that men who received oxytocin rated emotional faces with a higher level of dominance than men in the placebo condition [66].

Oxytocin administration also attenuated the effects of aversive conditioning by reducing the effect of rating the sympathetic level of emotional faces after receiving a shock. Thus, men who received oxytocin after an electric shock rated faces as more sympathetic than the placebo group [55]. Curiously, another experiment concluded that stress sweat odour interferes with the interpretation of facial expressions. In fact, the results showed that, in men and women, this odour led participants to interpret ambiguous facial expressions as fearful. Nonetheless, this fearful interpretation disappeared after oxytocin administration [52].

When the authors considered emotional valence, they concluded that all the participants (both men and women) presented shorter reaction times to disgusted, sad, and angry faces after oxytocin administration [67,96]. Nevertheless, two studies that only included women concluded that, after oxytocin administration, healthy women and women with borderline disorder (BD) presented shortened reaction times to angry and happy faces [76,95]. Similarly, the administration of this hormone entailed shortened reaction times to facial expressions in men [71], specifically for happy and fearful faces [32,69]. Additionally, men with social anxiety disorder (SAD) with high attachment avoidance who received oxytocin presented higher reaction times to disgusted and neutral faces than men with low attachment avoidance who received oxytocin [83].

Healthy participants, specifically men, and BD patients of both genders showed an initial avoidance of angry faces and approach tendencies toward happy faces, but these tendencies disappeared after drug administration [58,78]. That is, differences in the approach-avoidance tendencies disappeared depending on the emotional valence of the faces. Another study found that men characterized by social anxiety presented an initial attention bias toward processing threat cues in emotional faces, but this effect disappeared after receiving oxytocin, with their scores being similar to the healthy group [79].

It is interesting that, after oxytocin administration, healthy participants of both genders spent more time observing the eye region, regardless of the emotional valence of faces [40]. However, the consideration of all the faces involved increased gaze duration toward the eyes for both happy and angry faces in healthy participants [33,82]. Furthermore, major depressive disorder (MDD) patients of both genders who received oxytocin showed decreased attention to angry faces, but more attention to happy faces [82]. Patients with schizophrenia also experienced an increased fixation time on the eyes after receiving oxytocin [77,93]. 

The processing of facial emotional expressions incorporating interference emotional stimuli revealed that participants of both genders presented higher switch costs for sad faces, but not for angry faces. Nonetheless, this effect disappeared after drug administration [36]

Finally, we would like to take into account how drug administration interfered with emotion coding abilities for emotional facial expressions. In fact, several authors concluded that, after receiving oxytocin, a group of young men reported better long-term memory of happy facial expressions, but without affecting neutral and/or angry faces [43]. Furthermore, Savaskan et al. [60] concluded that memory recognition for emotional faces improved after drug administration in both men and women.

### 3.2. Cortisol

Regarding participants’ characteristics in the 16 articles that assess the role of cortisol in facial emotional processing, the number of participants ranged from 18 to 105. Moreover, the age of these individuals ranged from 18 to 60, and most of them were young adults (20–35 years old). Regarding gender distribution, 50% of these research studies assessed hormonal effects in groups of men and women. The other 44% exclusively included men, and 6% of these studies based their conclusions only on women (Table 3). 

Furthermore, most of the studies were based on healthy populations (75%), and a smaller percentage were conducted in clinical populations, such as SAD [112,113], posttraumatic stress disorder (PTSD) [110], and major depressive disorder (MDD) [111]. Lastly, all the manuscripts employed oral administration of hydrocortisone, except Dierolf et al. [99], which included intravenous drug injections.

Of all the studies included in our systematic review of the effects of cortisol manipulation on facial emotional processing (Table 4), only two of them found a significant main “hormone/drug” effect [104,110]. Although a significant “hormone” effect was not found in the rest of the manuscripts, the interaction between this variable and “emotional valence” or other variables (e.g., gender, group…) guaranteed that 56% of the studies reached statistical significance in interfering in facial emotional processing [98,99,100,102,103,104,105,107,108,111].

Regarding emotional accuracy, women seemed to discriminate angry faces better than men in placebo conditions, but the administration of hydrocortisone and subsequent increases in cortisol meant that “gender” differences in accurately recognizing angry and sad faces disappeared [100].

When focusing on attention to facial processing, results showed that hydrocortisone administration shortened reaction times on a facial emotional go/no-go task in PTSD patients and in healthy individuals of both genders [110]. That is, cortisol increases diminished attention-switch costs in processing emotional stimuli. Moreover, the administration of hydrocortisone seemed to cause men and women to present faster responses (shorter reaction times) to angry and fearful faces in comparison with other facial expressions [102,104], but when these faces were preceded by a distractor stimulus, participants of both genders showed slower responses (longer reaction times) on angry faces [102]. These effects were accentuated in participants with low basal cortisol levels and a minimum dose of hydrocortisone ranging from 4 to 10 mg [99,107]. Participants with high anxiety levels experienced shorter reaction times for fearful faces after drug administration [104], but men characterized by low anxiety presented initial longer reaction times during the presentation of fearful faces that disappeared after drug administration [105].

Two studies with healthy individuals (men and women) divided participants according to their tendency to confront emotional situations. In fact, in men characterized by a tendency to avoid emotional situations, hydrocortisone administration was related to an accentuated tendency to avoid all facial emotional expressions [108]. Furthermore, if women and men presented high provocation, hydrocortisone increased reaction times on all kinds of facial expressions [98]. Finally, this alteration in reaction times is specific to the healthy population (men and women) because participants with MDD of both genders did not experience variations after cortisol manipulation through “drug” administration [111].

Regarding memory processing of facial expressions, a study revealed that when the authors considered the “emotional valence” and participants’ “gender” along with “drug” administration, they concluded that hydrocortisone administration improved long-term memory only for angry faces, particularly in men [103].

### 3.3. Testosterone

Twelve articles assessed the effect of the manipulation of endogenous levels of testosterone on facial emotional processing (Table 5). The number of participants in the studies ranged from 16 to 117, and their ages ranged from 20 to 40 years old. It is important to highlight that there is a definite bias in the gender distribution of the sample. 

Thus, 85% of the studies employed women, and only 15% were exclusively composed of men [114,119]. The percentage of studies that assessed the healthy population reached 92%, with only one manuscript assessing the role of this hormone in people with SAD [126]. Finally, 15% of the studies employed topical administration of this hormone [114,119], and two others used nasal administration [124,125], whereas the rest of the manuscripts employed sublingual administration (85%).

Only two of the articles reported a significant main effect of “hormone” [117,123]. After the inclusion of a second variable interacting with the “drug” condition, 46% of the manuscripts reached statistical significance [114,118,120,121,122,126].

In women, a single administration of exogenous testosterone reduced the general ability to decode emotions when employing only the eye region of facial emotional expressions [123], angry faces [122], or trustworthiness in facial expressions [117]. Furthermore, they maintained attention to angry faces for more time [120], and they paid less attention to fearful faces after testosterone administration [121].

Regarding the individuals’ approach to or avoidance of the emotional valence of faces (Table 6), after testosterone administration, women experienced a reduction in the avoidance of angry faces [118]. This tendency to approach angry faces after testosterone administration obtained in the healthy population was also replicated in women with social anxiety disorder [126].

## 4. Discussion

The results described in our review highlight that, a priori, oxytocin, cortisol, and testosterone were not the main or only modulators of facial emotional processing. Only 18% of the articles presented an initial main effect of hormone manipulation, and after considering additional variables, around 53% of the manuscripts reached significance. In any case, it is clear that these hormones are involved in facial emotional processing, but conclusions should be tempered regarding their role as a potential treatment for psychiatric disorders or when attributing proneness to prosocial and/or antisocial behaviours to increases or decreases in the levels of these hormones. In fact, it is necessary to consider other hormones and neurotransmitters to establish and clarify the impact of the above-mentioned hormones on facial emotional processing. Therefore, based on the current data, we can qualify the evidence about hormones and facial emotional processing as inconclusive or even conflicting.

The main objective of this review was to summarize the effect of manipulating specific hormones such as oxytocin, cortisol, and testosterone in facial emotional processing. We strongly believe that the establishment of rigid inclusion criteria focused on robust experimental designs strengthened the value of this systematic review. Additionally, a similar pattern of significant results emerged for the three hormones. That is, a low percentage of studies presented a main effect of “hormone administration”, with differences emerging between groups (hormone vs. placebo) after considering the role of specific variables such as emotional valence and/or participants’ gender. Most of these results were obtained in healthy young adults (from 18 to 35) of both genders (from Western countries). Although this fact was important for their replicability, it should also be kept in mind that it limited the external validity of the conclusions, thus reinforcing the need to conduct this kind of research with heterogeneous samples.

Based on significant results and focusing on accuracy in decoding facial expressions of emotions, we can conclude that oxytocin increases tended to improve this ability [30,38,49,56,60,61,70,73,75,87], whereas cortisol and testosterone increases diminished it [100,123]. Moreover, after a high dose of oxytocin, certain individuals with schizophrenia and antisocial personality disorder showed an enhancement of their ability to accurately recognize facial emotions and they spent more time processing facial stimuli [85,96], and emotional biases in processing facial stimuli were even reduced in MDD patients [82].

Based on the aforementioned results, it would be suitable to hypothesize that the three hormones might interact inversely through hormonal receptors in specific brain areas to facilitate or inhibit accuracy in facial emotional processing. In this regard, it has been suggested that the occipital face area, PFC, anterior cingulate cortex (ACC), supplementary motor area, hypothalamus, superior temporal gyrus, fusiform gyrus, inferior frontal gyrus, periaqueductal grey, and/or amygdala, among others, are critical in facial emotional processing [127,128,129]. Curiously, there is enough evidence in human studies to support the presence of receptors for these three hormones in several of these brain structures [40,127,130,131,132]. Even so, nasal administration of oxytocin stimulates the olfactory bulb, which is directly connected to the amygdala via the piriform cortex [23,24,25,88]. Therefore, to interpret these behavioural results, it is important to pay attention to how amygdala activation varies subsequent to oxytocin or other hormone administration. In fact, whereas the administration of this hormone in healthy individuals reduced the activation of the amygdala in processing facial stimuli [34,89], testosterone and cortisol increased activation of this region [125]. Moreover, cortisol administration also modulated dmPFC activation in processing facial stimuli, increasing in women but decreasing in men [102] and affecting amygdala and hippocampus functioning [133]. When focusing on functional connectivity, oxytocin increases connectivity between the amygdala and the ACC and dmPFC [134]. However, testosterone administration reduced the amygdala-orbitofrontal PFC connectivity [124].

Before continuing, we think it is particularly important to highlight the need to be cautious about the role of cortisol and testosterone because it is not the case that the whole fraction of free cortisol and testosterone circulating in the blood interferes in cognition during human adulthood. In fact, corticosteroid-binding globulin inactivates free cortisol in the brain, and aromatization of testosterone to oestradiol is completely necessary for this hormone to interfere in cognitive processing. Therefore, it would be important to consider this when interpreting mutual interactions between hormones in facial emotional processing [135]. In the same way, there is extant research concluding that cortisol effects on the amygdala are mediated by noradrenaline and glucose, with their presence being necessary as mediators in emotional processing [135]. Furthermore, most of the manuscripts included which assess cortisol and testosterone employed oral administration, which delays the active fraction of hormones reaching the brain. Moreover, it was extremely difficult to track which brain structures were affected by these hormones.

As mentioned above, we might be tempted to assume that oxytocin tends to improve accurate recognition of all facial emotions, whereas cortisol or testosterone reduces it. Nevertheless, this statement is far from conclusive, especially if we analyse other variables. These effects would be facilitated by interfering in the activation of specific brain structures, mentioned above, and modifying their patterns of connectivity. Moreover, the consideration of other variables such as the emotional valence and the participants’ gender considerably increased the difficulty of interpretation, particularly in the case of oxytocin. In this regard, this hormone improved accuracy in decoding neutral, happy, and surprised facial expressions if we consider men and women together in a group [29,48,53,61,62,70,72]. Nonetheless, increases in this hormone reduced accuracy for fearful faces [48,49,84]. However, if we only consider men, accuracy for fearful faces improved after oxytocin improvements, but it decreased for angry faces [49]. Conversely, increases in this hormone enhanced women’s accuracy for angry faces [72]. Regarding cortisol, increases in cortisol were related to the disappearance of gender differences in discriminating angry and sad faces [100]. Hence, it might be possible to establish that there is a somewhat opposite effect between oxytocin and cortisol in terms of their effectiveness in discriminating among certain facial expressions, but not all of them. Nevertheless, as the data show, this statement is inaccurate.

In the case of rating the intensity (arousal) of emotional faces, whereas a study concluded that oxytocin administration increases perceived intensity but diminishes accuracy in decoding the valence of all the emotions [27], another study indicated that oxytocin diminished accuracy in decoding facial emotions, especially at low intensities, specifically for fear and happiness [65]. Conversely, Quintana et al. [57] concluded that oxytocin diminished perceived intensity, but only for anger. Although these results were based on healthy participants, it seems that results with clinical populations were congruent for variations in the perceived intensity of emotions, given that oxytocin administration increased perceived intensity of all six emotions in polydipsic patients [85] and the intensity of happiness in men with SAD [94]. However, Goldman et al. [85] also pointed out that patients experienced improvements in accuracy in decoding emotions. Hence, future research should consider differentiating between the two emotional stimuli (emotional valence and rating intensity or arousal) variables because they did not seem to be equally affected by hormonal manipulation, especially oxytocin.

Focusing on the reaction speed to facial stimuli, after oxytocin administration, participants (both genders together) experienced a reduction in their reaction times to happy faces [32,33,71,76,95], although long-term memory improved for all kinds of emotional stimuli [43,60]. They also spent more time looking at angry faces [33,82]. Conversely, the increase in oxytocin levels after exogenous administration also meant that participants of both genders showed shorter reaction times, for example, for disgusted, angry, and sad faces [67,96]. After dividing the groups according to participants’ gender, women experienced shortened reaction times to angry faces [76,95], but men showed shortened times for fearful faces [32,69].

On the one hand, cortisol diminished the reaction speed to any kind of facial emotional stimulus, particularly in women with high levels of hostility [102,104,110]. However, its administration did not alter processing speed in MDD patients, which seems logical because these patients often report alterations in hypothalamic–pituitary–adrenal axis regulation [111]. On the other hand, testosterone administration meant that women spent more time attending to angry faces, but they also dedicated less time to fearful faces [118,120,121,126]. Therefore, we cannot conclude that there was a specific general response for each hormone that altered the speed in accurately identifying and decoding facial emotions and was similar for all kinds of emotions.

On long-term memory, oxytocin and cortisol seem to modulate memory processes in opposite ways because oxytocin increases promote better long-term memory for happy faces [43], but cortisol does so for angry faces, especially in men [103]. In contrast, increases in oxytocin and cortisol meant that attention-switch costs disappeared after the increase in each hormone separately respectively [36,110]. Thus, when we consider other subprocesses related to facial emotional processing, such as processing speed and attention, we cannot describe a clear pattern for each hormone.

In any case, it should be highlighted that most of the studies included in this review were gender biased. Although a few of them combined both genders, the rest only included one gender. For example, testosterone studies contained mostly women. Combining the two would help to understand whether gender is related to different hormonal effects on emotional processing. Hence, future research should consider combining both genders instead of conducting studies based exclusively on men or women. We cannot understand the hormones’ role in terms of being clear facilitators or inhibitors of accuracy without examining their interactions with other factors. In fact, it would be necessary to incorporate their relationship with other neurotransmitter systems, for example, serotonin or dopamine, to clarify how they interfere with these emotional processes. This can be concluded because the PFC, hypothalamus, fusiform gyrus, inferior frontal gyrus, and/or amygdala, among others, are rich in receptors for both neurotransmitters [136,137]. Furthermore, empirical research included in this review pointed out that administration of oxytocin, cortisol, and/or testosterone also interfered with activation of some of the structures involved in processing emotional stimuli [34,40,117,127]. Thus, future research should consider how these hormones interact with the two neurotransmitters mentioned above in emotional processes. This would allow us to provide a broader and richer model to explain emotional processing, instead of developing simplistic and reductionist models.

Before ending this manuscript, we would like to mention how hormonal manipulation interferes with participants’ decisions in terms of approaching or avoiding certain types of emotional stimuli. There was some evidence showing that humans tend to present approach behaviours towards positive stimuli (e.g., happy or surprised faces) and avoidance of negative emotions (e.g., sad, angry, fearful…), but this “normal” tendency disappeared after oxytocin administration, even in BD patients [58,78]. Moreover, attention-switch costs also disappeared in patients with anxiety disorders and healthy individuals when processing emotional facial expressions [79]. This tendency was similar for testosterone. The manipulation of this hormone meant that women, both healthy and with anxiety disorders, experienced a reduction in their basal tendency to avoid angry faces [118]. Nonetheless, cortisol increases accentuated avoidance of facial emotional expressions, but not in all individuals because this effect was exclusively present in individuals who normally avoid emotional stimuli [126]. In addition, the importance of task relevance in approaching-avoiding decisions in the context of facial emotional stimuli must also be addressed [138,139,140]. In fact, it cannot be concluded that hormones automatically lead to one decision or another without considering whether the stimuli are relevant to the participant’s goals, given that, when this is the case, such stimuli seem to produce a reliable behavioural effect. Therefore, this variable should be incorporated in future research.

Some studies have examined how a spray with synthetic testosterone might be employed as a good method to modify endogenous levels. However, most of the studies conducted with testosterone and cortisol have employed oral (sublingual), topical, and/or intravenous (parenteral) administration of these hormones [118,121,123,126]. Accordingly, administration through these pathways increased the corresponding free fraction in the bloodstream, later traversing the blood brain barrier and, consequently, affecting the brain and specifically modulating facial emotional processing. However, depending on the administration route, the percentage of the exogenous hormone that reaches the central nervous system and the moment of the appearance of its effects vary [116]. Thus, it is necessary to monitor the route and the time, assessing their effects.

Although all the studies included in this review presented a robust design, there are several potential limitations that force us to temper the interpretation of the results. First, most of the studies presented a limited sample size and focused (see Table 1, Table 3, and Table 5) mainly on healthy young adults, and this was especially true in oxytocin studies. In fact, older populations should be examined because testosterone tends to decline with age, and cortisol regulation tends to dysregulate. Hence, it is difficult to generalize the results because these studies were based on relatively homogeneous samples (Western populations), and clinical populations with facial emotional processing alterations were underrepresented in these studies (e.g., individuals with personality disorders, mental disorders…). Second, it is also important to highlight that a significant effect of the drug emerged after dividing the initial sample into subgroups, thus increasing type 1 error. Therefore, it is highly likely that some significant results were false positives, particularly those near 0.05. However, some studies included the Bonferroni correction for multiple comparisons. Moreover, it is possible that the results in several manuscripts overlap because they are provided by the same research teams. Third, there were other methodological problems that should be clarified. Much of the research assessing emotional accuracy revealed that, for example, the eyes test presented poor internal consistency [141], which might explain its low sensitivity to detecting small changes in accuracy after drug administration. Hence, it is necessary to develop robust tools to measure this, or even review currently existing tools. Even though the face-task and eyes test presented good test-retest reliability after one year, it seemed that a learning effect might interfere with the scores when the effect of the drug was assessed after a few days and/or weeks. It is also important to highlight that significant brain connectivity and/or activation emerged after hormone manipulation in several manuscripts, but these studies failed to find significant behavioural performance differences [17,34,77,115]. A possible explanation would be the questionable robustness and sensitivity of these tools to detect changes. It is also surprising that researchers did not consider the effect of learning on tests employed to assess facial emotional processing. Lastly, none of the manuscripts included in this review assessed how the three hormones interact and interfere with emotional processing, thus increasing the difficulty of drawing conclusions. Therefore, we tried to assess them based on additional references included in the discussion. Additionally, other hormones should be considered, such as vasopressin and other sexual hormones, to offer a broader hormonal representation. These hormones, along with others, are closely related to the hormones presented in this review. Unfortunately, a low number of studies assessed their relationship with facial emotional processing in controlled designs with human samples. Thus, we removed other hormones during the initial stages of this review.

Finally, we might be tempted to hypothesize that oxytocin manipulation could be considered a potential therapeutic method for individuals who present poor empathic abilities, including emotional decoding deficits. Nevertheless, the current evidence does not support this idea, confirming that only a small percentage of studies obtain a main “hormone” effect. Moreover, their effects on emotional accuracy were subtle and even reversible. Other non-invasive behavioural treatments (e.g., cognitive training) have shown their robustness in promoting relatively stable empathic improvements. In the past, testosterone was proposed as the main cause of violence, but later studies confirmed its “modulator” role during adulthood, integrated in a more complex biological system. This would be like considering oxytocin to be a prosocial hormone by itself. Current evidence relativizes its role, relating it to prosocial and antisocial behaviours. Hence, it would be important to consider hormones’ role, but also including other biological markers and their interactions, as we did in this manuscript.

## 5. Conclusions

In sum, the present review demonstrated that specific hormones, such as oxytocin, cortisol, and testosterone, are involved in emotional decoding processes. However, their effects on this process seem to have been overestimated because less than 20% of the manuscripts included in this review reported a main “drug” effect. Hormonal manipulation reached significance in approximately 50% of the manuscripts after including emotional valence and participants’ gender. In any case, based on current evidence (e.g., brain structures rich in specific hormonal receptors, activated to process facial and/or emotional stimulus) and the studies included in this review, we propose a brain circuity that might regulate facial emotional processing. Obviously, we did not forget several neurotransmitters (e.g., dopamine, serotonin…) systems that might interfere with and modulate the associations between these hormones in explaining several important cognitive processes involved in emotion decoding abilities (e.g., accuracy, speed processing, attention, eye gaze, memory…). We also presented limitations of the studies assessing this topic, helping to improve future research designs and guide potential targets in emotion processing. Hence, knowing more about the hormonal factors that affect this ability would favour not only our comprehension, but also the development of potential treatments or therapies to improve this ability. In fact, this knowledge would make it possible to target which emotional processing factors might be altered, in order to develop more effective intervention strategies.

## Figures and Tables

**Table 1:** Main characteristics of each study that assessed oxytocin’s role in facial emotional processing in healthy and clinical populations (in alphabetical order).

Authors	Sample	Age, Gender, Handedness	Dose	Way Administrat	Time	Task	Research Design
***Healthy Population***							
Campbell et al. [26]	68 68	72.07 ± 6.49 19.68 ± 1.79; 50% ♂ 50% ♀ -	20 IU OX	Single administration Nasal	45 min	Facial emotion recognition task	Double-blind, randomized, placebo-controlled
Cardoso et al. [27]	82	From 18 to 30; 49% ♂ 51% ♀ -	24 IU OX	Single administration Nasal	120 min	Mayer-Salovey-Caruso emotional intelligence test (face task)	Double-blind, randomized, placebo-controlled
Colonnello et al. [28]	84	25.00 ± 2.00; 100% ♂ -	24 IU OX	Single administration Nasal	50 min	Facial emotion recognition task	Double-blind, randomized, placebo-controlled
Di Simplicio et al. [29]	29	From 18 to 30; 100% ♂ -	24 IU OX	Single administration Nasal	50 min	Facial expression recognition task and Cambridge face memory test	Double-blind, randomized, placebo-controlled
Domes et al. [30]	30	25.3 ± 2.2; 100% ♂ -	24 IU OX	Single administration Nasal	45 min	Reading the mind in the eyes test	Double-blind, placebo-controlled
Domes et al. [31]	16	24.2 ± 2.5; 100% ♀ Right-handed	24 IU OX	Single administration Nasal	45–60 min	Facial emotion recognition task	Double-blind, placebo-controlled
Domes et al. [32]	69	24.0 ± 3.1; 100% ♂ -	24 IU OX	Single administration Nasal	45 min	Facial emotion recognition task	Double-blind, randomized, placebo-controlled
Domes et al. [33]	62	24.0 ± 2.5; 100% ♂ -	24 IU OX	Single administration Nasal	40 min	Dynamic affect recognition evaluation	Double-blind, randomized, placebo-controlled
Domes et al. [34]	30	25.7 ± 2.91; 100% ♂ -	24 IU OX	Single administration Nasal	45 min	Implicit facial affect recognition paradigm	Double-blind, randomized, placebo-controlled
Ellenbogen et al. [35]	102	From 18 to 35; 50% ♂ 50% ♀ -	24 IU OX	Single administration Nasal	45 min	Negative affective priming task	Double-blind, randomized, placebo-controlled
Ellenbogen et al. [36]	57	From 18 to 35; 48% ♂ 52% ♀ -	24 IU OX	Single administration Nasal	45 min	Modified spatial cueing task	Double-blind, randomized, placebo-controlled
Ellingsen et al. [37]	39	26 years; 49% ♂ 51% ♀ Right-handed	40 IU OX	Single administration Nasal	40 min	Facial emotion recognition task	Double-blind, placebo-controlled
Feeser et al. [38]	82	27.9 ± 4.7; 100% ♂ -	24 IU OX	Single administration Nasal	45 min	Karolinska directed emotional faces	Double-blind, randomized, placebo-controlled
Fischer-Shofty et al. [39]	27	26.93 ± 3.51; 100% ♂ -	24 IU OX	Single administration Nasal	45 min	Facial emotion recognition task	Double-blind, randomized, placebo-controlled
Gamer et al. [40]	46	25.0 ± 3.7; 100% ♂ Right-handed	24 IU OX	Single administration Nasal	45 min	Emotion classification paradigm	Double-blind, placebo-controlled
Gamer & Büchel. [41]	38	24.6 ± 3.5; 100% ♂ Right-handed	24 IU OX	Single administration Nasal	45 min	Facial emotion recognition task	Double-blind, placebo-controlled
Grainger et al. [42]	118	From 18 to 90; 47% ♂ 53% ♀ -	24 IU OX	Single administration Nasal	90 min	Facial trust stimuli	Double-blind, randomized, placebo-controlled
Guastella et al. [43]	69	19.98 ± 2.27; 100% ♂ -	24 IU OX	Single administration Nasal	45 min	Facial emotion recognition task	Double-blind, randomized, placebo-controlled
Hirosawa et al. [44]	20	31.4 years; 100% ♂; Right-handed	24 IU OX	Single administration Nasal	45 min	Facial emotion recognition task	Single-blind, placebo-controlled
Hoge et al. [45]	47	43.3 ± 10.7; 62% ♂ 38% ♀ -	30 IU OX	Single administration Nasal	25 min	Affective misattribution task	Double-blind, randomized, placebo-controlled
Horta et al. [46]	48 54	22.4 ± 3.0; 52% ♂ 48% ♀ - 71.2 ± 4.9; 44% ♂ 56% ♀ -	24 IU OX	Single administration Nasal	90 min	Dynamic facial emotion identification task	Double-blind, randomized, placebo-controlled
Hubble et al. [47]	40	20.98 ± 4.55; 100% ♂ -	24 IU OX	Single administration Nasal	30 min	Facial emotion recognition task	Double-blind, randomized, placebo-controlled
Leknes et al. [48]	39	From 20 to 39; 49% ♂ 51% ♀ Right-handed	40 IU OX	Single administration Nasal	45 min	Facial emotion recognition task	Double-blind, placebo-controlled
Lischke et al. [49]	47	26.09 ± 3.41; 100% ♂ -	24 IU OX	Single administration Nasal	45 min	Facial emotion recognition task	Double-blind, randomized, placebo-controlled
Luo et al. [50]	86	22.41 ± 2.054; 50% ♂ 50% ♀ Right-handed	24 IU OX	Single administration Nasal	45 min	Chinese facial affective picture system	Double-blind, randomized, placebo-controlled
Lynn et al. [51]	40	44.00 ± 10.32; 60% ♂ 40% ♀ -	30 IU OX	Single administration Nasal	50 min	Facial emotion recognition task	Double-blind, placebo-controlled
Maier et al. [52]	50	24.54 ± 3.09; 48% ♂ 52% ♀ Right-handed	40 IU OX	Single administration Nasal	30 min	Forced-choice emotional face recognition task	Double-blind, randomized, placebo-controlled
Marsh et al. [53]	50	From 20 to 40; 58% ♂ 42% ♀ -	24 IU OX	Single administration Nasal	35 min	Facial emotion recognition task	Double-blind, randomized, placebo-controlled
Perry et al. [54]	30	38.9 ± 10.6; 63% ♂ 37% ♀ -	24 IU OX	Single administration Nasal	45 min	The face-context composites	Double-blind, randomized, placebo-controlled
Petrovic et al. [55]	30	From 19 to 40; 100% ♂ Right-handed	32 IU OX	Single administration Nasal	45 min	Affective ratings in response to presentation of faces	Double-blind, randomized, placebo-controlled
Prehn et al. [56]	47	-; 100% ♂ -	24 IU OX	Single administration Nasal	45 min	Dynamic facial emotion recognition task	Double-blind, randomized, placebo-controlled
Quintana et al. [57]	16	From 18 to 35; 100% ♂ -	8, 24 IU OX 1 IU (blood)	Single administration Nasal or intravenously	40 min	Facial emotion recognition task	Double-blind, randomized, placebo-controlled
Radke et al. [58]	24	21.46 ± 1.93; 100% ♂ -	24 IU OX	Single administration Nasal	45 or 65 min	Approaching-avoiding face task	Double-blind, randomized, placebo-controlled
Riem et al. [59]	50	19.62 ± 1.47; 100% ♀ -	16 IU OX	Single administration Nasal	60 min	Reading the mind in the eyes test	Double-blind, randomized, placebo-controlled
Savaskan et al. [60]	36	27.5 ± 1.3; 50% ♂ 50 ♀ -	20 IU OX	Single administration Nasal	30 min	Facial emotion recognition task	Single-blind, randomized, placebo-controlled
Schulze et al. [61]	56	24.18 ± 3.12; 100% ♂ -	24 IU OX	Single administration Nasal	45 min	Facial emotion recognition task	Double-blind, randomized, placebo-controlled
Shin et al. [62]	37	23.1 ± 2.8; 100% ♂ -	40, 32 IU OX	Single administration Nasal	45 min	Facial emotion recognition task	Double-blind, randomized, placebo-controlled
Skvortsova et al. [63]	88	21.5 ± 2.4; 100 ♀ -	24 IU OX	Single administration Nasal	50 min	Facial emotion recognition task	Single-blind, randomized, placebo-controlled
Skvortsova et al. [64]	9	21 average; 100% ♀ -	24 IU OX	Single administration Nasal	50 min	Facial attractiveness and trustworthiness task	Single-blind, randomized, placebo-controlled
Spengler et al. [65]	116	24.7 ± 4.4; 100% ♂ -	12, 24, 48 OX	Single administration Nasal	45 min	Facial emotion recognition task	Double-blind, randomized, placebo-controlled
Teed et al. [66]	20	-; 100% ♂ -	24 IU OX	Single administration Nasal	45 min	Facial emotion recognition task	Double-blind, placebo-controlled
Theodoridou et al. [67]	120	22.4 years; 50% ♂ 50% ♀ -	24 IU OX	Single administration Nasal	35 min	Facial emotion recognition task	Double-blind, randomized, placebo-controlled
Thienel et al. [68]	37	From 23 to 26; 100% ♂ -	24 IU OX	Single administration Nasal	40 min	Face rating task	Double-blind, placebo-controlled
Tollenaar et al. [69]	20	21 ± 3; 100% ♂ -	24 IU OX	Single administration Nasal	35 min	Emotional gaze cueing task	Double-blind, randomized, placebo-controlled
Xu et al. [70]	60	From 19 to 27; 100% ♂ Right-handed	40 IU OX	Single administration Nasal	45 min	Social dual-target rapid serial visual presentation task	Double-blind, randomized, placebo-controlled
Xu et al. [71]	71	21.85 ± 0.32; 100% ♂ -	24 IU OX	Single administration Nasal	45 min	The antisaccade paradigm	Double-blind, randomized, placebo-controlled
Yue et al. [72]	87	21.2 ± 1.76; 49% ♂ 51% ♀ -	24 IU OX	Single administration Nasal	45 min	Emotional face working memory task	Double-blind, randomized, placebo-controlled
***Clinical Population***							
Averbeck et al. [73]	21 SZ	38.2 ± 1.8; 100% ♂ -	24 IU OX	Single administration Nasal	50 min	Hexagon emotion discrimination task	Double-blind, placebo-controlled
Bach et al. [74]	18 AUD 15 controls	From 18 to 65; 100% ♂ Right-handed	24 IU OX	Single administration Nasal	45 min	Matching shape or face task	Double-blind, placebo-controlled
Bate et al. [75]	10 DP 10 controls	49.2 ± 14.2; 70% ♂ 30% ♀; 80% Right-handed 46.8 ± 13.2; -	24 IU OX	Single administration Nasal	45 min	Cambridge face memory test and Cambridge face perception test	Double-blind, randomized, placebo-controlled
Bertsch et al. [76]	40 BD 41 controls	From 18 to 36; 100% ♀ -	26 IU OX	Single administration Nasal	45 min	Emotion classification task	Double-blind, randomized, placebo-controlled
Bradley et al. [77]	33 SZ 39 controls	40.3 ± 15.5 39.8 ± 13.7 100% ♂ -	40 IU OX	Single administration Nasal	50 min	Facial emotion recognition task	Double-blind, randomized, placebo-controlled
Brüne et al. [78]	13 BPD 13 controls	28.6 ± 7.22 25.7 ± 6.76; 31% ♂ 69% ♀ -	24 IU OX	Single administration Nasal	45 min	Emotional dot probe task	Double-blind, randomized, placebo-controlled
Clark-Elford et al. [79]	16 SAD 26 controls	27.13 ± 9.25; 100% ♂ -	24 IU OX	Single administration Nasal	45 min	Emotional dot probe task	Double-blind, randomized, placebo-controlled
Davis et al. [80]	27 SZ	37.0 ± 10.8 42.8 ± 9.1; 100% ♂ -	40 IU OX	Single administration Nasal	30 min (+1 week, +1 month)	Facial emotion recognition task	Double-blind, placebo-controlled
Davis et al. [81]	23 SZ	From 18 to 56; 100% ♂ -	40 IU OX	Single administration Nasal	30 min	Facial emotion recognition task	Double-blind, placebo-controlled
Domes et al. [82]	43 MDD	47 years; 42% ♂ 58% ♀ -	24 IU OX	Single administration Nasal	45 min	Emotional dot probe task	Double-blind, randomized, placebo-controlled
Fang et al. [83]	60 SAD	24.39 years; 100% ♂ -	24 IU OX	Single administration Nasal	45 min	Modified Posner task	Double-blind, randomized, placebo-controlled
Fischer-Shofty et al. [84]	31 SZ 35 controls	31.8 ± 6.53 29.49 ± 5.59 83% ♂ 17% ♀ -	24 IU OX	Single administration Nasal	45 min	FaceMorphing task	Double-blind, randomized, placebo-controlled
Goldman et al. [85]	13 SZ 11 controls	53 ± 3 44 ± 9 38 ± 13 45%♂ 55% ♀ -	10, 20 IU OX	Three administrations Nasal	45 min	Facial emotion recognition task	Double-blind, placebo-controlled
Gorka et al. [86]	16 GSAD 17 controls	29.8 ± 9.1 29.9 ± 10.5 100% ♂; Right-handed	24 IU OX Placebo	Single administration Nasal	45 min	Emotional face matching task	Double-blind, randomized, placebo-controlled
Guastella et al. [87]	16 ASD	14.88 ± 2.42; 100% ♂ -	18 and 24 IU OX	Single administration Nasal	45 min	Reading the mind in the eyes test-revised	Double-blind, randomized, placebo-controlled
Guastella et al. [88]	50 ASD	From 12 to 18; 100% ♂ -	18 IU and 24 IU OX	Daily for 4–8 weeks Nasal	4 weeks	Reading the mind in the eyes test	Double-blind, placebo-controlled
Labuschagne et al. [89]	18 GSAD	From 18 to 55; 100% ♂ Right-handed	24 IU OX	Single administration Nasal	45 min	Emotional face matching task	Double-blind, randomized, placebo-controlled
Labuschagne et al. [90]	18 GSAD 18 controls	29.4 ± 9.0 29.9 ± 10.2 100% ♂; Right-handed	24 IU OX	Single administration Nasal	45 min	Computerized emotional face processing task	Double-blind, placebo-controlled
Mitchell et al. [91]	32 AUD	28.9 ± 7.15; 59% ♂ 41% ♀ -	50 IU OX	Single administration Nasal	45 min	Reading the mind in the eyes test	Double-blind, randomized, placebo-controlled
Pedersen et al. [92]	20 SZ	39.00 ±11.18 35.78 ±9.52 85% ♂ 15% ♀ -	24 IU OX	Daily for 14 days Nasal	14 days	Trustworthiness task	Double-blind, randomized, placebo-controlled
Porffy et al. [93]	19 SZ	38.4 ± 7.3; 100% ♂ Right-handed	40 IU OX	Single administration Nasal	120 min	Free-viewing task	Double-blind, placebo-controlled
Quintana et al. [94]	17 SAD	From 18 to 35; 100% ♂ -	8 or 24 IU OX 1 IU (blood)	Single administration Nasal	40 min	Emotional dot probe task	Double-blind, randomized, placebo-controlled
Schneider et al. [95]	114 BD	From 18 to 52; 100% ♀ -	24 IU	Single administration Nasal	75 min	Approach–avoidance task	Double-blind, randomized, placebo-controlled
Timmermann et al. [96]	22 ASPD 29 controls	24.2 ± 4.1; 63% ♂ 37% ♀ -	24 IU OX	Single administration Nasal	45 min	Emotion classification paradigm	Double-blind, placebo-controlled
Woolley et al. [97]	29 SZ 31 controls	44.6 ± 10.7 42.5 ± 14.1 ♂; -	40 IU OX	Single administration Nasal	30 min	Reading the mind in the eyes test	Double-blind, randomized, placebo-controlled

**Note.** ASD: autism spectrum disorder; ASPD: antisocial personality disorder; AUD: alcohol use disorder; BD: borderline disorder; DP: developmental prosopagnosia; GSAD: generalized social anxiety disorder; IU: international units; MDD: major depressive disorder; OX: oxytocin, SAD: social anxiety disorder; SZ: schizophrenia; -: non assessed; ♂: men; ♀: women.

**Table 2:** Main results for each study that included oxytocin manipulation (in alphabetical order for each dominion).

Authors	Accuracy	Reaction Time	Rating Arousal	Attention	Memory	Trustworthiness/ Friendliness	Dominance/ Hostility	Significant after Including…	Effect Size
**Healthy Population**									
Campbell et al. [26]	Ns	-	-	-	-	-	-	Hormone x Gender x Age (*p* = 0.014)	np^2^ = 0.05
Cardoso et al. [27]	Significant	-	Significant	-	-	-	-	-	np^2^ = 0.09
Colonnello et al. [28]	-	Ns	-	-	-	-	-	Ns	-
Di Simplicio et al. [29]	Ns	Ns	-	-	Ns	-	-	Hormone x Valence (*p* = 0.031)	np^2^ = 0.161
Domes et al. [30]	Significant	-	-	-	-	-	-	Ns	-
Domes et al. [31]	-	-	Ns	Ns	-	-	-	Ns	-
Domes et al. [32]	-	Ns	-	Ns	-	-	-	Hormone x Emotion (*p* = 0.05)	-
Domes et al. [33]	Ns	-	-	Ns	-	-	-	Hormone x Emotion (*p* = 0.015)	-
Domes et al. [34]	Ns	-	-	-	-	-	-	-	-
Ellenbogen et al. [35]	-	Ns	-	-	-	-	-	Ns	-
Ellenbogen et al. [36]	-	Ns	-	-	-	-	-	Hormone x Emotion (*p* < 0.05)	np^2^ = 0.08
Ellingsen et al. [37]	-	-	-	-	-	Ns	-	Hormone x Facial Expression x Touch (*p* = 0.025)	-
Feeser et al. [38]	Significant	-	-	-	-	-	-	-	-
Fischer-Shofty et al. [39]	Ns	Ns	-	-	-	-	-	Hormone x Emotion (*p* < 0.05)	-
Gamer et al. [40]	Ns	-	-	Ns	-	-	-	Hormone x Initial Fixation (*p* = 0.043)	-
Gamer & Büchel. [41]	Ns	-	-	-	-	-	-	Ns	-
Grainger et al. [42]	-	-	-	-	-	Ns	-	Ns	-
Guastella et al. [43]	-	-	-	-	Ns	Ns	-	Hormone x Emotion (*p* = 0.04)	-
Hirosawa et al. [44]	-	Ns	-	-	-	-	Ns	Ns	-
Hoge et al. [45]	-	-	-	-	-	Ns	-	Hormone x Gender (*p* < 0.048)	np^2^ = 0.118
Horta et al. [46]	Ns	Ns	-	-	-	-	-	Ns	-
Hubble et al. [47]	Ns	Ns	-	Ns	-	-	-	Ns	-
Leknes et al. [48]	Ns	-	-	-	-	-	-	Hormone x Task x Emotion (*p* < 0.05)	-
Lischke et al. [49]	Ns	-	-	Ns	-	-	-	Hormone x Emotion (*p* = 0.02)	-
Luo et al. [50]	Ns	-	Ns	-	-	-	-	Ns	-
Lynn et al. [51]	-	Ns	-	-	-	-	-	Hormone x Gender (*p* < 0.049)	np^2^ = 0.11
Maier et al. [52]	Ns	Ns	-	-	-	-	-	Hormone x Sweat x Interference (*p* < 0.025)	np^2^ = 0.11
Marsh et al. [53]	Ns	-	-	-	-	-	-	Hormone x Emotion (*p* < 0.05)	np^2^ = 0.06
Perry et al. [54]	Ns	-	-	-	-	-	-	Hormone x Emotion (*p* = 0.026)	-
Petrovic et al. [55]	-	Ns	Ns	-	-	-	-	Hormone x Shock (*p* < 0.05)	-
Prehn et al. [56]	Significant	-	-	-	-	-	-	-	np^2^ = 0.10
Quintana et al. [57]	-	-	Ns	-	-	Ns	-	Hormone x Emotion (*p* = 0.003)	-
Radke et al. [58]	-	Ns	-	-	-	-	-	Hormone x Emotion x Movements (*p* = 0.015)	np^2^ = 0.23
Riem et al. [59]	Ns	-	-	-	-	-	-	Hormone x Love Withdrawal (*p* = 0.01)	-
Savaskan et al. [60]		-	-	-	Significant	-	-	-	-
Schulze et al. [61]	Significant	-	-	-	-	-	-	-	np^2^ = 0.128
Shin et al. [62]	Ns	Ns	-	-	-	-	-	Hormone x Emotion (*p* = 0.01) (dose 40 IU)	np^2^ = 0.64
Skvortsova et al. [63]	-	-	Ns	-	-	-	-	Ns	-
Skvortsova et al. [64]	-	-	-	-	-	Ns	-	Ns	-
Spengler et al. [65]	Ns	-	Ns	-	-	-	-	Hormone x Emotion x Dose x Time (*p* = 0.03)	np^2^ = 0.08
Teed et al. [66]	-	-	-	-	-	Ns	Ns	Hormone x Condition (*p* = 0.020)	np^2^ = 0.045
Theodoridou et al. [67]	-	Ns	-	-	-	-	-	Hormone x Emotion (*p* < 0.05)	-
Thienel et al. [68]	-	-	-	-	-	Ns	-	Hormone x Sexual Orientation x Emotion x State (*p* < 0.03)	-
Tollenaar et al. [69]	-	-	-	Significant	-	-	-	-	np^2^ = 0.25
Xu et al. [70]	Significant	-	-	-	-	-	-	-	np^2^ = 0.138
Xu et al. [71]	-	-	-	Significant	-	-	-	-	np^2^ = 0.10
Yue et al. [72]	Ns	-	-	-	Ns	-	-	Hormone x Task x Emotion (*p* < 0.05)	np^2^ = 0.09
**Clinical Population**									
Averbeck et al. [73]	Significant	-	-	-	-	-	-	-	-
Bach et al. [74]	Ns	-	Ns		-	-	-	Ns	-
Bate et al. [75]	Significant	-	-	-	Significant	-	-	-	np^2^ = 0.426
Bertsch et al. [76]	-	Ns	-	-	-	-	-	Hormone x Emotion x Fixation (*p* = 0.03)	-
Bradley et al. [77]	Ns	-	-	-	-	-	-	Hormone x Group (*p* < 0.001)	-
Brüne et al. [78]	-	Ns	-	-	-	-	-	Hormone x Emotion x Cognition x Group (*p* = 0.03)	-
Clark-Elford et al. [79]	-	Ns	-	-	-	-	-	Hormone x Group (*p* < 0.01)	np^2^ = 0.22
Davis et al. [80]	Ns	-	-	-	-	-	-	Ns	-
Davis et al. [81]	Ns	-	-	-	-	-	-	Ns	-
									
Domes et al. [82]	Ns	Ns	-	-	-	-	-	Hormone x Emotion (*p* = 0.014)	np^2^ = 0.139
Fang et al. [83]	-	Ns	-	-	-	-	-	Hormone x Attachment x Emotion (*p* < 0.05)	-
Fischer-Shofty et al. [84]	Ns	-	-	-	-	-	-	Hormone x Emotion (*p* = 0.028)	np^2^ = 0.077
Goldman et al. [85]	Ns	-	Ns	-	-	-	-	Hormone x Dose x Group (*p* < 0.01)	-
Gorka et al. [86]	Ns	Ns	-	-	-	-	-	Ns	
Guastella et al. [87]	Significant	-	-	Ns	-	-	-	-	-
Guastella et al. [88]	Ns	-	-	-	-	-	-	Ns	-
Labuschagne et al. [89]	Ns	-	-	-	-	-	-	Ns	
Labuschagne et al. [90]	Ns	Ns	-	-	-	-	-	Ns	
Mitchell et al. [91]	Ns	Ns	-	-	-	-	-	Hormone x Difficulty (*p* = 0.04)	-
Pedersen et al. [92]	-	-	-	-	-	Ns	-	Ns	
Porffy et al. [93]	-	-	-	Significant	-	-	-	-	-
Quintana et al. [94]	Ns	-	Ns	-	-	-	-	Hormone x Dose x Emotion (*p* = 0.02)	d = 0.63
Schneider et al. [95]	-	Ns	-	-	-	-	-	Hormone x Emotion (*p* = 0.014)	np^2^ = 0.06
Timmermann et al. [96]	Ns	Ns	-	-	-	-	-	Hormone x Group x Emotion (*p* = 0.023)	np^2^ = 0.08
Woolley et al. [97]	-	-	-	Ns	-	-	-	Hormone x Group x Difficulty (*p* = 0.03)	

**Note.** Ns: non-significant; -: non assessed; np^2^: partial eta squared; d: Cohen’s d.

**Table 3:** Main characteristics of each study that assessed cortisol’s role in facial emotional processing in healthy and clinical populations (in alphabetical order).

Authors	Sample	Age, Gender, Handedness	Dose	Way Administrat	Time	Task	Research Design
**Healthy Population**							
Bertsch et al. [98]	56	From 19 to 25; 50% ♂ 50% ♀ Right-handed	20 mg hydrocortisone	Single administration Oral	1 h	Emotional Stroop task	Double-blind, placebo-controlled
Dierolf et al. [99]	38	23.00 ± 2.89; 100% ♂ Right-handed	4 mg hydrocortisone	Single administration Intravenously	2 min	Emotion–gender task switch	Double-blind, randomized, placebo-controlled
Duesenberg et al. [100]	75	24.5 ± 3.4; 49% ♂ and 51%♀ -	10 mg hydrocortisone	Single administration Oral	45 min	Facial emotion recognition task	Double-blind, randomized, placebo-controlled
Henckens et al. [101]	72	21 years; 100% ♂ Right-handed	10 mg hydrocortisone	Single administration Oral	1 h 15 or 4 h 45 min	Dynamic facial expression task	Double-blind, randomized, placebo-controlled
Ma et al. [102]	40	22.8 ± 5.4; 50% ♂ and 50%♀ Right-handed	100 mg hydrocortisone	Single administration Oral	2 h	Shifted-attention emotion appraisal task	Double-blind, randomized, placebo-controlled
Putman et al. [103]	18	From 18 to 23; 100% ♂ -	40 mg hydrocortisone	Single administration Oral	2 h	Face relocation task	Double-blind, counterbalanced, placebo-controlled
Putman et al. [104]	20	20.1 average; 100% ♂ -	40 mg hydrocortisone	Single administration Oral	1 h 15 min	Masked emotional Stroop task	Double-blind, counterbalanced, placebo-controlled
Putman et al. [105]	20	From 18 to 23; 100% ♂ Right-handed	40 mg hydrocortisone	Single administration Oral	45 min	Emotional gaze cueing task	Double-blind, placebo-controlled
Schwabe et al. [106]	80	23.53 ± 0.34; 50% ♂ and 50%♀; Right-handed	20 mg hydrocortisone	Single administration Oral	45 min	Rating fearfulness in facial expressions	Double-blind, randomized, placebo-controlled
Taylor et al. [107]	64	From 19 to 43; 22% ♂ 78% ♀ -	10 mg or 40 mg hydrocortisone	Single administration Oral	1 h	Negative affective priming task	Double-blind, randomized, placebo-controlled
van Peer et al. [108]	40	From 18 to 30; 100% ♂ Right-handed	50 mg hydrocortisone	Single administration Oral	1 h 15 min	Approach–avoidance task	Double-blind, randomized, placebo-controlled
Vasa et al. [109]	32	26.63 ± 4.30; 50% ♂ 50% ♀ -	0.5 mg/kg hydrocortisone	Single administration Blood	30 min	Emotional dot probe task	Double-blind, randomized, placebo-controlled
**Clinical Population**							
Carvalho Fernando et al. [110]	64 PTSD	>18 years; 100% ♀ Right-handed	10 mg hydrocortisone	Single administration Oral	45 min	Emotional go/no-go paradigm	Double-blind, randomized, placebo-controlled
Schlosser et al. [111]	104 MDD	From 18 to 60; 38% ♂ 62% ♀ -	10 mg hydrocortisone	Single administration Oral	45 min	Emotional go/no-go paradigm	Double-blind, randomized, placebo-controlled
van Peer et al. [112]	17 SAD	31.4 ± 10.0; 100% ♂ Right-handed	50 mg hydrocortisone	Single administration Oral	2 h 30 min	Emotional Stroop task	Double-blind, randomized, placebo-controlled
van Peer et al. [113]	20 SAD	32.8 ± 10.2; 45% ♂ 55%♀ Right-handed	50 mg hydrocortisone	Single administration Oral	1–2 h	Approach–avoidance task	Double-blind, randomized, placebo-controlled

**Note.** MDD: major depressive disorder; PTSD: posttraumatic stress disorder; SAD: social anxiety disorder; -: non assessed; ♂: men; ♀: women.

**Table 4:** Main results for each study that included cortisol manipulation (in alphabetical order for each dominion).

Authors	Accuracy	Interference	Memory	Reaction Time	Rating Arousal	Attention	Significant after Including…	Effect Size
**Healthy Population**								
Bertsch et al. [98]	-	-	-	Ns	-	-	Hormone x Group (*p* = 0.005)	np^2^ = 0.19
Dierolf et al. [99]	Ns	-	-	Ns		-	Hormone x Cue x Emotion x Task Switch (*p* < 0.05)	ω^2^ = 0.04
Duesenberg et al. [100]	Ns	-	-	-	-	-	Hormone x Gender x Emotion (difficulty) (*p* = 0.009)	-
Henckens et al. [101]	-	-	-	Ns	-	-	Ns	-
Ma et al. [102]	-	-	-	Ns	-	-	Hormone x Emotion (*p* = 0.032)	-
Putman et al. [103]	-	-	Ns	-	-	-	Hormone x Emotion (*p* = 0.006)	-
Putman et al. [104]	-	Significant	-	-	-	-	-	np^2^ = 0.234
Putman et al. [105]	-	-	-	-	-	Ns	Hormone x Emotion x Anxiety levels (*p* = 0.053)	np^2^ = 0.193
Schwabe et al. [106]	-	-	-	-	Ns	-	Ns	-
Taylor et al. [107]	-	-	-	Ns	-	-	Hormone x Emotion (*p* < 0.05)	-
van Peer et al. [108]	Ns	-	-	Ns	-	-	Hormone x Group x Arm movement (*p* < 0.0001)	np^2^ = 0.29
Vasa et al. [109]	-	Ns	-	Ns	-	-	Ns	-
**Clinical Population**								
Carvalho et al. [110]	-	-	-	Significant	-	-	-	np^2^ = 0.06
Schlosser et al. [111]	-	-	-	Ns	-	-	Hormone x Group (*p* = 0.034)	-
van Peer et al. [112]	-	-	-	Ns	-	-	Ns	-
van Peer et al. [113]	-	-	-	Ns	-	-	Ns	-

**Note.** Ns: non-significant; -: non assessed; np^2^: partial eta squared; ω^2^: omega squared.

**Table 5:** Main characteristics of each study that assessed testosterone’s role in facial emotional processing in healthy and clinical populations (in alphabetical order).

Authors	Sample	Age, Gender, Handedness	Dose	Way Administrat	Time	Task	Research Design
**Healthy Population**							
Bird et al. [114] Study 1	30	21.21 ± 2.19; 100% ♂; -	150 mg of AndroGel	Single administration Topical administration	50% (2 h) 50% (4 h)	Facial ratings of trustworthiness task	Double-blind, randomized, placebo-controlled
Bird et al. [114] Study 2	117	25.27 ±8 4.98; 100% ♂; -	150 mg of AndroGel	Single administration Topical administration	2 h 45 min	Facial ratings of dominance task	Double-blind, placebo-controlled
Bos et al. [115]	16	20.8 ± 2.0; 100% ♀; Right-handed	0.5 mg of testosterone	Single administration Sublingual	4 h	Reading the mind in the eyes test	Double-blind, randomized, placebo-controlled
Bos et al. [116]	16	20.8 ± 2.0; 100% ♀; Right-handed	0.5 mg of testosterone	Single administration Sublingual	4 h	Facial rating of trustworthiness task	Randomized, counterbalanced, placebo-controlled
Bos et al. [117]	24	20.02; 100% ♀; -	0.5 mg of testosterone	Single administration Sublingual	4 h	Facial rating of trustworthiness task	Double-blind, counterbalanced design, placebo-controlled
Enter et al. [118]	24	29 ± 8.4; 100% ♀; Right-handed	0.5 mg of testosterone	Single administration Sublingual	4 h 30 min	Approach-avoidance task	Double-blind, randomized, placebo-controlled
Goetz et al. [119]	16	From 18- 44; 100% ♂; Right-handed	100 mg of AndroGel	Single administration Topical administration	50 min	Emotional face matching task	Double-blind, counterbalanced, placebo-controlled
Terburg et al. [120]	20	From 20 to 25; 100% ♀ -	0.5 mg of testosterone	Single administration Sublingual	4 h	Social-dominance task	Placebo-controlled, counterbalanced
van Honk et al. [121]	16	From 19 to 26; 100% ♀ Right-handed	0.5 mg of testosterone	Single administration Sublingual	4 h	Masked emotional Stroop task	Double-blind, randomized, placebo-controlled
van Honk & Schutter, [122]	16	From 19 to 26; 100% ♀ Right-handed	0.5 mg of testosterone	Single administration Sublingual	4 h	Emotion-recognition task	Double-blind, randomized, placebo-controlled
van Honk et al. [123]	16	21 years; 100% ♀ -	0.5 mg of testosterone	Single administration Sublingual	4 h	Reading the mind in the eyes test	Double-blind, placebo-controlled
van Wingen et al. [124]	25	42 years; 100% ♀ Right-handed	0.9 mg of testosterone	Single administration Nasal dose	45 min	Face emotion recognition task	Double-blind, randomized, placebo-controlled
van Wingen et al. [125]	44	From 19 to 50; 100% ♀ Right-handed	0.9 mg of testosterone	Single administration Nasal dose	45 min	Face emotion recognition task	Double-blind, placebo-controlled
**Clinical Population**							
Enter et al. [126]	17 SAD	22.8 ± 5.0; 100% ♀ Right-handed	0.5 mg of testosterone	Single administration Sublingual	4 h 30 min	Approach-avoidance task	Double-blind, placebo-controlled

**Note.** SAD: social anxiety disorder; -: non assessed; ♂: men; ♀: women.

**Table 6:** Main results for each study that included testosterone manipulation (in alphabetical order for each dominion).

Authors	Accuracy	Interference	Reaction Time	Trustworthiness/Friendliness	Dominance/Hostility	Significant after Including…	Effect Size
**Healthy Population**							
Bird et al. [114] Study 1	Ns	-	Ns	Ns	-	Hormone x Order administration (*p* = 0.006)	np^2^ = 0.242
Bird et al. [114] Study 2	-	-	-	-	Ns	Ns	-
Bos et al. [115]	Ns	-	Ns	-	-	Ns	-
Bos et al. [116]	-	-	-	Ns	-	Ns	-
Bos et al. [117]	-	-	-	Significant	-	-	-
Enter et al. [118]	-	-	Ns	-	-	Hormone x Emotion (*p* = 0.033)	np^2^ = 0.05
Goetz et al. [119]	Ns	-	Ns	-	-	Ns	-
Terburg et al. [120]	Ns	-	-	-	-	Hormone x Emotion (*p* = 0.008)	np^2^ = 0.32
van Honk et al. [121]	Ns	-	-	-	-	Hormone x Emotion (*p* = 0.015)	-
van Honk & Schutter, [122]	Ns	-	-	-	-	Hormone x Threat Expression x Emotion (*p* < 0.05)	-
van Honk et al. [123]	Significant	-	-	-	-	-	-
van Wingen et al. [124]	Ns	-	Ns	-	-	Ns	-
van Wingen et al. [125]	Ns	-	Ns	-	-	Ns	-
**Clinical Population**							
Enter et al. [126]	-	-	Ns	-	-	Hormone x Emotion (*p* = 0.032)	np^2^ = 0.236

**Note.** Ns: non-significant; -: non assessed; np^2^: partial eta squared.

## Data Availability

Not applicable.
